# Interrogation Techniques and Interface Circuits for Coil-Coupled Passive Sensors

**DOI:** 10.3390/mi9090449

**Published:** 2018-09-09

**Authors:** Marco Demori, Marco Baù, Marco Ferrari, Vittorio Ferrari

**Affiliations:** Department of Information Engineering, University of Brescia, Via Branze, 38-25123 Brescia, Italy; marco.bau@unibs.it (M.B.); marco.ferrari@unibs.it (M.F.); vittorio.ferrari@unibs.it (V.F.)

**Keywords:** coil-coupled sensor, passive sensor unit, resonant sensor, telemetric sensor, distance-independent contactless interrogation

## Abstract

Coil-coupled passive sensors can be interrogated without contact, exploiting the magnetic coupling between two coils forming a telemetric proximity link. A primary coil connected to the interface circuit forms the readout unit, while a passive sensor connected to a secondary coil forms the sensor unit. This work is focused on the interrogation of sensor units based on resonance, denoted as resonant sensor units, in which the readout signals are the resonant frequency and, possibly, the quality factor. Specifically, capacitive and electromechanical piezoelectric resonator sensor units are considered. Two interrogation techniques, namely a frequency-domain technique and a time-domain technique, have been analyzed, that are theoretically independent of the coupling between the coils which, in turn, ensure that the sensor readings are not affected by the interrogation distance. However, it is shown that the unavoidable parasitic capacitance in parallel to the readout coil introduces, for both techniques, an undesired dependence of the readings on the interrogation distance. This effect is especially marked for capacitance sensor units. A compensation circuit is innovatively proposed to counteract the effects of the parasitic input capacitance, and advantageously obtain distance-independent readings in real operating conditions. Experimental tests on a coil-coupled capacitance sensor with resonance at 5.45 MHz have shown a deviation within 1.5 kHz, i.e., 300 ppm, for interrogation distances of up to 18 mm. For the same distance range, with a coil-coupled quartz crystal resonator with a mechanical resonant frequency of 4.432 MHz, variations of less than 1.8 Hz, i.e., 0.5 ppm, have been obtained.

## 1. Introduction

The ongoing downscaling of modern sensing devices is facing the main challenges of ensuring adequate power supply sources and removing wired connections. The power supply in wireless sensors has been traditionally provided by batteries that, however, have limited lifetime and need periodic recharge/replacement. Moreover, issues related to their degradation and the environmental impact for their disposal need to be considered.

As an alternative approach, energy harvesting techniques have gained increasing interest and undergone extensive investigations. Energy is harvested from the surroundings in the form of vibrations, motion, thermal energy, or solar energy, just to name a few. Suitable energy converters have been developed to transform the harvested energy into electrical energy using different principles, like piezoelectric [1,2], electromagnetic [3], thermoelectric [4] or pyroelectric [5,6] effects. Depending on the input source, the converted power can be sufficient to supply, continuously or intermittently, one or more sensing devices, which can transmit the measurement information through a radio frequency (RF) link to a receiving and supervising unit, thus creating a completely autonomous system without the need for power supply and cabling [7].

Alternatively, solutions based on the radio frequency identification (RFId) technologies can be adopted to implement sensing solutions exploiting electromagnetic coupling or RF fields to energize and transmit measurement information [8,9]. These solutions are typically based on low power configurations relying on a microcontroller to interface passive sensors, such as capacitive or resistive sensors [10]. Implantable sensors for medical analyses and monitoring are important examples where this solution can be advantageously applied [11,12,13].

Both energy harvesting and RFId systems use active electronics in the sensor unit which, in specific situations, can be a limitation, like in hostile, high-temperature, and chemically-harsh environments, where traditional silicon-based electronics cannot operate. In this context, the use of coil-coupled passive sensors, i.e., devices which do not need active components and integrated circuits to operate, is attractive. This solution exploits the magnetic coupling between a primary and a secondary coil to read passive sensors. The primary coil, along with the reading circuitry, forms the readout unit, which reads the sensor unit composed of the sensor element connected to the secondary coil [14,15,16,17]. This approach offers the promising advantage of reducing the cost of the passive sensor unit, allowing the production of disposable sensors, such as labels, with a passive sensor connected to the embedded coil [18,19].

In this paper, passive coil-coupled sensor units having a resonant behavior will be considered. The resonant behavior allows extracting the measurement information through the reading of the resonant frequency of the sensor unit [14,20]. This approach is robust because it is unaffected by the disturbances, such as noise and electromagnetic interferences, which typically affect the signal amplitude. Specifically, two kinds of sensors are investigated, as introduced in Section 2, namely, capacitive sensors, which form a resonant LC circuit with the secondary coil, and piezoelectric resonators, such as Quartz Crystal Resonators (QCRs) [21] or ceramic Resonant Piezo Layers (RPLs) [22].

One of the challenges of the contactless readout of passive sensors is to adopt reading techniques independent of the coupling between the primary and secondary coils [20,23]. This, in turn, would ensure that the readings are not affected by the interrogation distance. Two readout techniques, that are virtually independent of the coupling, are presented and discussed in detail in Section 3. In particular, a frequency-domain technique based on impedance measurements [20] and a time-domain technique called time-gated technique [21] are discussed. Both techniques suffer from significant accuracy degradation, due to the unavoidable parasitic capacitance in parallel to the readout coil that introduces a dependence of the readings on the interrogation distance. This undesirable effect is investigated in detail. Section 4 illustrates a compensation circuit that is innovatively proposed to counteract the effects of the parasitic input capacitance and advantageously obtain distance-independent readings in real operating conditions. Section 5 reports a set of experimental results on prototypes that successfully demonstrate the validity of the proposed approach and circuit.

## 2. Coil-Coupled Passive Sensors

**Figure 1:**
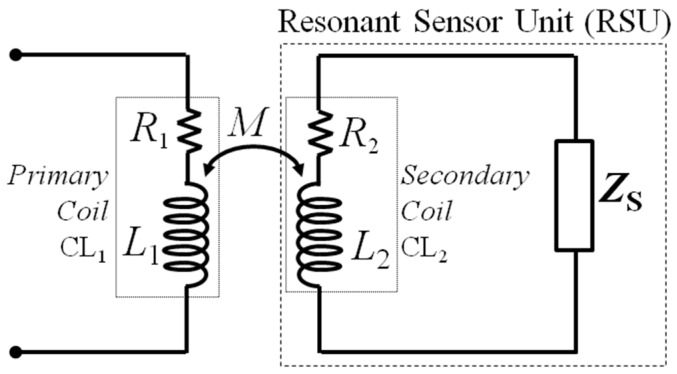
Equivalent circuit of a coil-coupled passive sensor.

A coil-coupled passive sensor is represented in its basic form by the schematic diagram of Figure 1. A primary coil CL_1_ with inductance *L*_1_ and series resistance *R*_1_ is magnetically coupled to the secondary coil CL_2_ with inductance *L*_2_ and resistance *R*_2_. The magnetic coupling is accounted for by the mutual inductance *M*, which depends on the geometry of *L*_1_ and *L*_2_ and their spatial arrangement. Alternatively, the magnetic coupling can be described through the coupling factor *k*, which is a nondimensional parameter defined as $k=M/\sqrt{\left(L_{1}L_{2}\right)}$, resulting in |*k*|≤1. In the following, the values of *L*_1_, *R*_1_ and *L*_2_, *R*_2_ will be considered as fixed, while the value of *M*, and hence *k*, can change due to variations of the distance or orientation between CL_1_ and CL_2_.

CL_2_ is connected to the generic impedance *Z*_S_, which models the sensing element. In the following, the relevant cases will be considered where *Z*_S_ either forms, with *L*_2_, a second order network with complex conjugate poles, i.e., *Z*_S_ is predominantly capacitive, or *Z*_S_ itself includes a second order network with complex conjugate poles, i.e., *Z*_S_ comprises an LCR network. In both cases, resonance can occur in the secondary circuit where the quantity to be sensed via *Z*_S_ influences the resonant frequency and, possibly, the damping. Therefore, the resulting combination will be termed Resonant Sensor Unit (RSU).

Importantly, for the RSU, the measurement information is carried by the frequency of the readout signal instead of its amplitude. The adoption of the resonant measuring principle has two main advantages with respect to amplitude-based techniques [24,25]. Firstly, the resonant principle is robust against external interferences or nonidealities that affect the signal amplitude. Secondly, as it will be illustrated in the following, the resonant principle, combined with suitable electronic techniques, can ensure that the readout frequency is made independent of the distance between CL_1_ and the RSU.

The present theory will consider two specific cases for *Z*_S_ and the resulting RSU.

**Figure 2:**
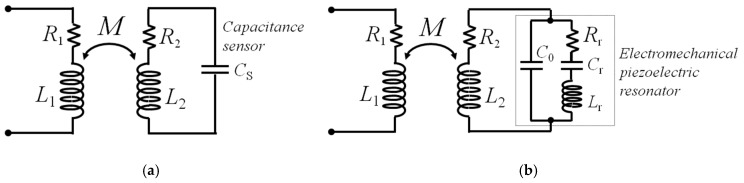
Equivalent circuits of the two considered cases for a coil-coupled resonant sensor unit (RSU): (**a**) capacitance sensor *C*_S_; (**b**) electromechanical piezoelectric resonator represented with its equivalent Butterworth–van Dyke (BVD) model.

In the first case, *Z*_S_ is a capacitance sensor of value *C*_S_, forming, with *L*_2_, an LC resonant circuit as shown in Figure 2a. The resonant frequency *f*_S_ and quality factor *Q*_S_ of the RSU are

(1) $$f_{S}=\frac{1}{2\pi \sqrt{L_{2}C_{S}}};\;Q_{S}=\frac{1}{R_{2}}\sqrt{\frac{L_{2}}{C_{S}}}.$$

In the second case, *Z*_S_ is the equivalent impedance of piezoelectric resonant sensors, like QCRs and RPLs. Their electromechanical behavior around resonance can be modelled with the Butterworth–van Dyke (BVD) equivalent lumped-element circuit, as shown in Figure 2b. The BVD circuit is composed of a motional, i.e., mechanical branch, and an electrical branch. The motional branch comprises the series of inductance *L*_r_, capacitance *C*_r_, and resistance *R*_r_, which respectively represent the equivalent mass, compliance, and energy losses of the resonator. The electrical branch is formed by the parallel capacitance *C*_0_, due to the dielectric material of the resonator. Under excitation by a voltage source, the mechanical resonant frequency *f*_r_, i.e., the frequency at which the current in the motional arm is maximum, corresponds to the series resonant frequency of the BVD circuit, i.e., the frequency at which the reactance of the mechanical branch impedance vanishes [26]. Accordingly, *f*_r_ and the quality factor *Q*_r_ of the electromechanical resonator can be expressed as

(2) $$f_{r}=\frac{1}{2\pi \sqrt{L_{r}C_{r}}};\;Q_{r}=\frac{1}{R_{r}}\sqrt{\frac{L_{r}}{C_{r}}}.$$

Typically, when electromechanical piezoelectric resonators are used as sensors, the measurand quantity generates variations of the parameters of the motional branch *L*_r_–*C*_r_–*R*_r_ and, as a consequence, of *f*_r_ and *Q*_r_.

## 3. Analysis of the Interrogation Techniques

### 3.1. General Considerations

Specific interrogation techniques are required to extract information from the RSU through electronic measurements at the primary coil, exploiting the advantage of coil-coupled, i.e., contactless, operation.

One major issue to consider is the dependence of the mutual inductance *M* and coupling factor *k* of the coils on geometrical parameters, such as their distance, alignment, and relative orientation. Techniques that are influenced by the value of *M*, or equivalently *k*, would require keeping such geometrical parameters fixed and constant [27,28]. On the other hand, in most practical applications, keeping the distance and the alignment between coils fixed is unpractical/unfeasible. Therefore, as a key requirement for out-of-the-lab use of coil-coupled sensors, robust measurement techniques are demanded that are independent of *k*.

In the following, two innovative techniques are illustrated to perform *k*-independent readout of RSUs of both capacitance and electromechanical piezoelectric resonator types. In particular, the first is a frequency-domain technique which relies on the measurement of the reflected impedance at CL_1_. The second is a time-domain technique, termed time-gated technique, which considers the free damped response of the RSU measured at the primary coil after that the RSU has been energized.

### 3.2. k-Independent Techniques Applied to Coil-Coupled Capacitance Sensors

**Figure 3:**
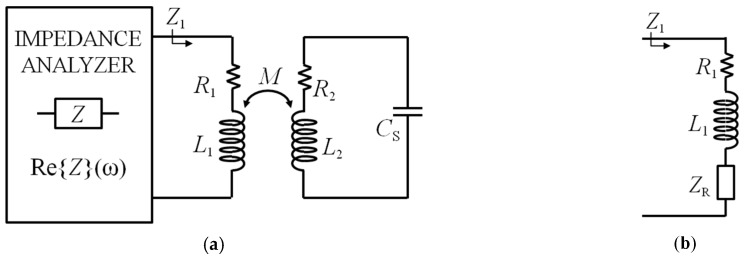
(**a**) Block diagram of the interrogation system based on impedance measurement from the primary coil; (**b**) equivalent circuit for the calculation of *Z*_1_.

Figure 3a shows the block diagram of the readout technique based on impedance measurements, where the readout system consists in an impedance analyzer connected to the primary coil CL_1_. From the equivalent circuit of Figure 3b, the impedance *Z*_1_, as a function of *ω* = 2π*f*, is

(3) $$Z_{1}=R_{1}+j\omega L_{1}+Z_{R}=R_{1}+j\omega L_{1}+\omega^{2}k^{2}L_{1}L_{2}\frac{1}{R_{2}+j\omega L_{2}+\frac{1}{j\omega C_{S}}}.$$

It can be seen from Equation (3) that the effect of the coupling with the RSU results in a reflected impedance *Z*_R_ in series with the primary coil that makes the total impedance *Z*_1_ dependent on the coupling factor *k*. Nevertheless, the resonant frequency *f*_S_ and the quality factor *Q*_S_ of the RSU, defined in Equation (1), can be obtained from the real part of *Z*_1_ [20], given by

(4) Re{Z1}(ω)=R1+ω2k2L1L2R2R22+(ωL2−1ωCS)2.

**Figure 4:**
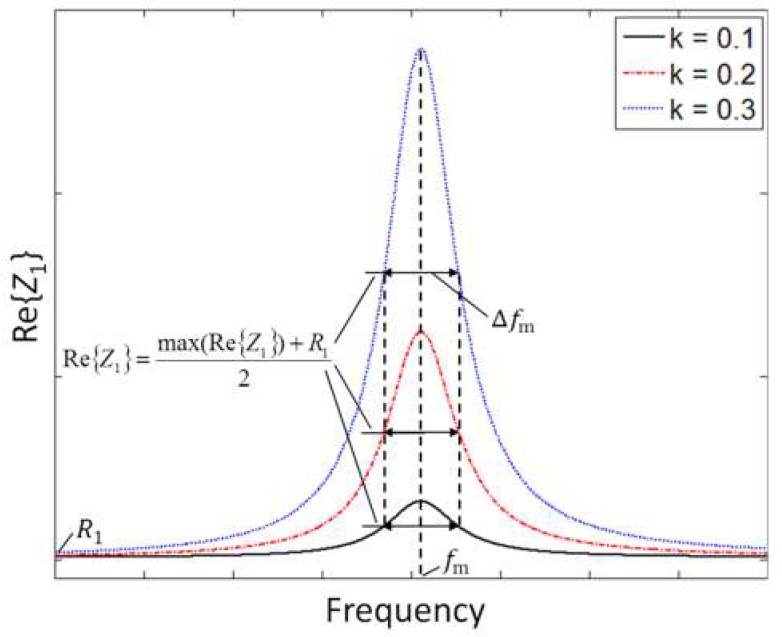
Real part of *Z*_1_ as a function of frequency from Equation (4) for three different values of *k*.

Re{*Z*_1_} has a local maximum at the frequency *f*_m_ = *ω*_m_/2π, which can be found by equating to zero the derivative of Equation (4) with respect to ω. Interestingly enough, *f*_m_ is independent of *k*, and it can be related to *f*_S_ and *Q*_S_ only. Then, combining Equations (1) and (4), the following relations hold:

(5) fm=f|max(Re{Z1})=2QS4QS2−2fS; QS≈fSΔfm,

where Δ*f*_m_ is the full width at half maximum (FWHM) of Re{*Z*_1_}, around *f*_m_ [20]. If *Q*_S_ is sufficiently large, then *f*_m_ ≈ *f*_S_, with a relative deviation |*f*_m_ − *f*_S_|/*f*_S_ < 100 ppm for *Q*_S_ > 50. Equations (4) and (5) demonstrate that from the measurement of *f*_m_ and Δ*f*_m_ in Re{*Z*_1_}, the frequency *f*_S_ and quality factor *Q*_S_ of the capacitive RSU can be advantageously extracted independently from *k*. Figure 4 shows sample plots of Re{*Z*_1_} calculated for three different values of *k*, and illustrates the definition of Δ*f*_m_*.* Consistently with Equation (4), *k* only affects amplitude.

**Figure 5:**
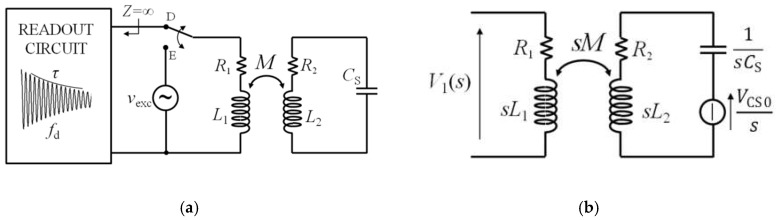
(**a**) Block diagram of the time-gated technique; (**b**) equivalent circuit of the time-gated technique during the detection phase.

The operating principle of the time-gated technique is shown in Figure 5a [21]. It comprises two subsequent alternating phases, namely, excitation and detection phases. During the excitation phase, when the switch is in the E position, CL_1_ is connected to the sinusoidal signal *v*_exc_(*t*) to excite the RSU through inductive coupling. During the subsequent detection phase, when the switch is in the D position, the excitation signal is disconnected, and CL_1_ is connected to a readout circuit with a high-impedance input, resulting in a virtually zero current in CL_1_.

The input voltage *v*_1_(*t*) of the readout circuit during the detection phase D can be derived by taking the inverse Laplace transform of the corresponding voltage *V*_1_(*s*), where *s* is the complex frequency. Since the RSU forms a second order LCR network, the voltage *v*_1_(*t*) is expected to be a damped sinusoid with frequency *f*_d_ and a decay time *τ*_d_ from which the resonant frequency *f*_S_ and the quality factor *Q*_S_ of the RSU can be inferred.

Generally, assuming that the detection phase D starts at *t* = 0, the readout voltage *v*_1_(*t*) depends on the initial conditions at *t* = 0 of all the reactive elements, namely *C*_S_, *L*_1_, *L*_2_, and *M*. The effect of the initial conditions on *v*_1_(*t*) for *t* > 0 is to globally affect only its starting amplitude, while the complex frequencies of the network, that define *f*_d_ and *τ*_d_, are unaltered. Therefore, without losing any generality, the single initial condition *V*_CS0_ defined as the voltage across *C*_S_ at *t* = 0 can be considered, neglecting the remaining ones. As an equivalent alternative that does not change the consequences of the present treatment, *V*_CS0_ can also be seen as an effective initial condition.

As a result, the equivalent circuit of Figure 5b representing the time-gated configuration during the detection phase in the Laplace domain can be considered, and the expression of *V*_1_(*s*) is

(6) $$V_{1}\left(s\right)=k\sqrt{\frac{L_{1}}{L_{2}}}V_{\mathrm{CS}0}\frac{s}{s^{2}+s\frac{R_{2}}{L_{2}}+\frac{1}{L_{2}C_{S}}}.$$

The corresponding time expression *v*_1_(*t*) can be calculated:

(7) $$v_{1}\left(t\right)=k\sqrt{\frac{L_{1}}{L_{2}}}\sqrt{\frac{4Q_{s}^{2}}{4Q_{s}^{2}-1}}V_{\mathrm{CS}0}e^{-\frac{t}{\tau_{d}}}\cos\left[2\pi f_{d}t-\mathrm{atan}\left(\frac{1}{2\pi f_{d}\tau_{d}}\right)\right].$$

The signal *v*_1_(*t*) is a damped sinusoid with damped frequency *f*_d_ and decay time *τ*_d_ that are related to *f*_S_ and *Q*_S_ of the RSU as

(8) $$f_{d}=f_{S}\sqrt{1-\frac{1}{4Q_{S}{}^{2}}};\;\tau_{d}=\frac{Q_{S}}{\pi f_{S}}.$$

**Figure 6:**
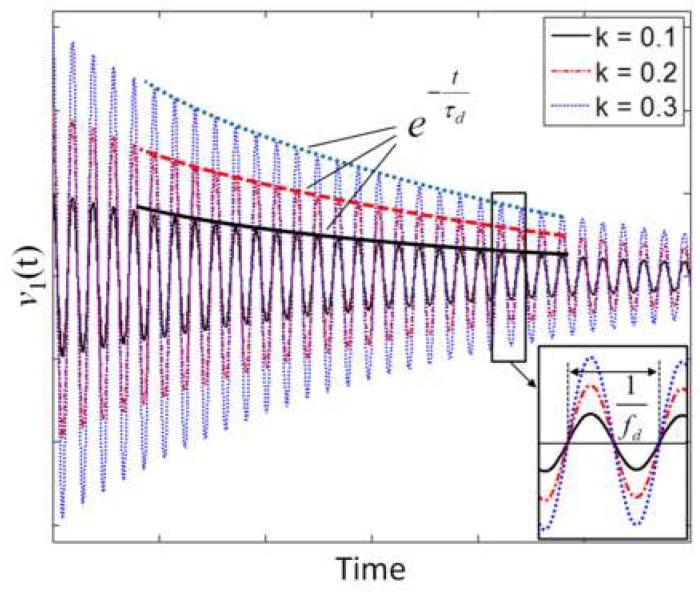
Voltage *v*_1_(t) during the detection phase calculated for three different values of the coupling factor *k.*

If *Q*_S_ is sufficiently large, it results in *f*_d_ ≈ *f*_S_, with a relative deviation |*f*_d_ − *f*_S_|/*f*_S_ < 50 ppm for *Q*_S_ > 50. Notably, the coupling factor *k* only acts as an amplitude factor on *v*_1_(*t*) without influencing either *f*_d_ or *τ*_d_. Figure 6 reports sample plots of *v*_1_(*t*) calculated for three different values of *k*.

In summary, Equations (7) and (8) demonstrate that, under the assumptions made, the time-gated technique can also allow extraction of the frequency *f*_S_ and quality factor *Q*_S_ of the capacitive RSU, independently of *k.*

### 3.3. k-Independent Techniques Applied to Coil-Coupled Electromechanical Piezoelectric Resonators

Considering the technique based on impedance measurements with reference to the equivalent circuit of Figure 2b, the impedance *Z*_1_ measured at the primary coil can be expressed as

(9) $$Z_{1}=R_{1}+j\omega L_{1}+\omega^{2}k^{2}L_{1}L_{2}\frac{1}{R_{2}+j\omega L_{2}+\frac{1}{j\omega C_{0}}||\left(j\omega L_{r}+\frac{1}{j\omega C_{r}}+R_{r}\right)}.$$

As it can be observed in Equation (9), the impedance *Z*_1_ depends on the coupling factor *k*. Nevertheless, also in this case, the frequency *f*_r_ can be extracted from the frequency of the maximum of the real part of *Z*_1_.

**Figure 7:**
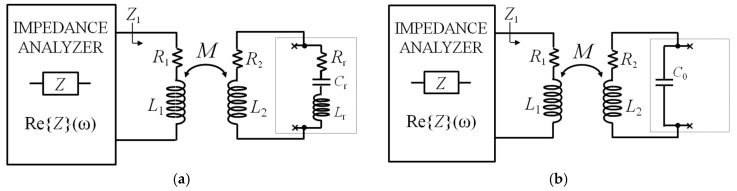
(**a**) Block diagram of the interrogation system with equivalent circuit of electromechanical piezoelectric resonator around *f*_r_; (**b**) block diagram of the interrogation system with equivalent circuit of electromechanical piezoelectric resonator for *f* >> *f*_r_.

Close to the angular frequency *ω*_r_ = 2π*f*_r_, the impedance of the motional arm Z_r_ = *R*_r_ + *jωL*_r_ + 1/(*jωC*_r_) has a magnitude typically much smaller than that of the impedance of *C*_0_, i.e., |Z_r_| << 1/*ωC*_0_. Then, the presence of *C*_0_ can be neglected, resulting in the simplified equivalent circuit of Figure 7a. Accordingly, Re{*Z*_1_} around *ω*_r_ has the following approximated expression:

(10) Re{Z1}≈R1+ω2k2L1L2Rr+R2(Rr+R2)2+[ω(Lr+L2)−1ωCr]2.

Equation (10) has the same form as Equation (4) and, hence, Re{*Z*_1_} has a maximum at the frequency *f*_m_r_ given by

(11) $$f_{m\_r}=f_{r2}\frac{2Q_{r2}}{\sqrt{4Q_{r2}{}^{2}-2}},\;\mathrm{where}\;f_{r2}=\frac{1}{2\pi \sqrt{\left(L_{r}+L_{2}\right)C_{r}}}\;\mathrm{and}\;Q_{r2}=\frac{1}{R_{r}+R_{2}}\;\sqrt{\frac{L_{r}+L_{2}}{C_{r}}}.$$

It can be observed that for large *Q*_r2_, *f*_m_r_ ≈ *f*_r2_ with a deviation |*f*_m_r_ − *f*_r2_|*/f*_r2_ < 100 ppm for *Q*_r2_ > 50. In addition, assuming that *L*_2_ << *L*_r_, the frequency *f*_r2_ approximates *f*_r_ and, hence, *f*_m_r_ ≈ *f*_r_ holds. Similarly, if *R*_2_ << *R*_r_, *Q*_r2_ approaches *Q*_r_. Importantly, again, the coupling factor *k* acts only as an amplitude factor that advantageously does not affect either the frequency or the quality factor of the resonance.

Considering, now, the frequencies *ω* >> *ω*_r_, the impedance magnitude of *C*_0_ is smaller than the impedance magnitude of *Z*_r_, which then can be neglected, obtaining the equivalent circuit of Figure 7b. Consequently, the following approximated expression of Re{*Z*_1_} results:

(12) Re{Z1}≈R1+ω2k2L1L2R2R22+(ωL2−1ωC0)2.

Also Equation (12) has the same form as Equation (4), and it can be seen that Re{*Z*_1_} now has a maximum at the frequency *f*_m_el_:

(13) $$f_{m\_\mathrm{el}}=f_{\mathrm{el}}\frac{2Q_{\mathrm{el}}}{\sqrt{4Q_{\mathrm{el}}{}^{2}-2}},\;\mathrm{where}\;=f_{\mathrm{el}}=\frac{1}{2\pi \sqrt{L_{2}C_{0}}}\;\mathrm{and}\;Q_{\mathrm{el}}=\frac{1}{R_{2}}\sqrt{\frac{L_{2}}{C_{0}}}.$$

From the previous analysis, it can be concluded that Re{*Z*_1_} has two peaks: the first is related to the mechanical resonance *f*_r_, the second to the electrical resonance *f*_el_. With the previous assumptions on the values of *L*_r_ and *L*_2_, and considering that, typically, *C*_r_ << *C*_0_, then it follows that *f*_el_ >> *f*_r_.

**Figure 8:**
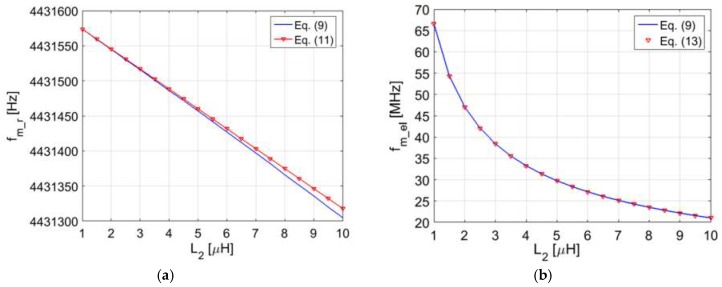
(**a**) Comparison of *f*_m_r_ derived from the maximum of Re{*Z*_1_} for frequencies around *f*_r_, in Equation (9), and the approximate value from Equation (11) as a function of *L*_2_; (**b**) comparison of *f*_m_el_ derived from the maximum of Re{*Z*_1_} for *f* >> *f*_r_, in Equation (9), and the approximate value from Equation (13) as a function of *L*_2_.

To validate, numerically, the proposed approximations, Figure 8a,b report the comparison of the values of *f*_m_r_ and *f*_m_el_ derived respectively from Equations (11) and (13), and the frequency of the maxima derived numerically from Re{*Z*_1_} in Equation (9) as a function of *L*_2_. The following values of the BVD model of a 4.432 MHz AT-cut QCR have been used: *C*_0_ = 5.72 pF, *R*_r_ = 10.09 Ω, *L*_r_ = 77.98 mH, and *C*_r_ = 16.54 fF. For CL_1_ and CL_2_, the values of the electrical parameters are *L*_1_ = 8.5 µH, *R*_1_ = 5 Ω, and *R*_2_ = 5 Ω.

Figure 8a shows that for *L_2_* up to 10 µH, the values of *f*_m_r_ predicted from Equation (11) are within 3 ppm with respect to the numerical solutions from Equation (9). Additionally, for the same range of variation of *L*_2_, a remarkable agreement is obtained between *f*_m_el_ predicted from Equation (13) and the numerical solution.

The possibility to interrogate coil-coupled electromechanical piezoelectric resonators with the time-gated technique independently from the coupling has been previously demonstrated [21].

**Figure 9:**
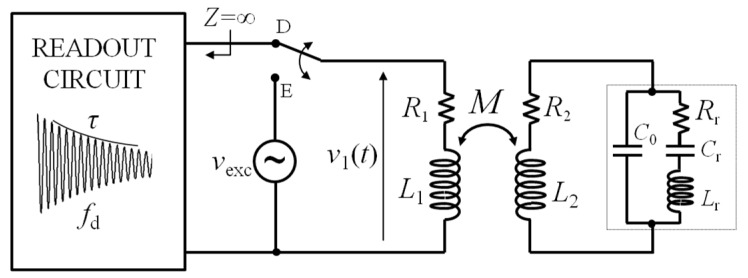
Block diagram of the time-gated technique applied to a coil-coupled electromechanical piezoelectric resonator.

The RSU configuration of Figure 9 has been studied in [21], showing that the open circuit voltage *v*_1_(*t*) at CL_1_ during the detection phase, after the RSU has been energized in the excitation phase, is the sum of two damped sinusoids: one at frequency *f*_d_r_ with exponential decaying time *τ*_r_, and one at frequency *f*_d_el_ with exponential decaying time *τ*_el_.

The damped sinusoid at *f*_d_r_ is due to the mechanical response of the resonator, while the one at *f*_d_el_ is due to the electrical response of *L*_2_ that interacts with the electrical capacitance *C*_0_. In addition, for suitable values of *L*_2_ and *R*_2_, and considering the typical values of the equivalent parameters of the BVD model of a QCR, the decaying time *τ*_r_ is orders of magnitude larger than *τ*_el_. Thus, the damped sinusoid at frequency *f*_d_el_ decays to zero much faster than the damped sinusoid at frequency *f*_d_r_. Hence, the former can be neglected in the expression of *v*_1_(*t*), which results in

(14) $$v_{1}\left(t\right)≅k\sqrt{L_{1}L_{2}}A_{r}e^{-\frac{t}{\tau_{r}}}\cos\left(2\pi f_{d\_r}t+\theta_{r}\right)-\delta \left(t\right)L_{1}i_{L1}\left(0\right),$$

where the amplitude and phase coefficients *A*_r_ and *θ*_r_ are functions of both the initial conditions at the beginning of the detection phase (*t* = 0), and the electrical and mechanical parameters of the system. The last term represents the contribution of the initial current *i_L_*_1_(0) in the primary inductor. From Equation (14), it can be seen that *k* acts only as a scaling factor for the amplitude of *v*_1_, without affecting the sensor response parameters *f*_d_r_ and *τ*_r_. From a simplified analysis that considers the undamped system with *R*_2_ = 0 and *R*_r_ = 0, under the hypothesis that (*ωC*_0_)^−1^ >> *ωL*_2_ at the frequency *f*_r_ and that *Q*_r_ is large, it has been obtained that the frequency *f*_d_r_ can be approximated with the following relation:

(15) $$f_{d\_r}\approx f_{r}\left(1-\frac{1}{2}\frac{L_{2}}{L_{r}}\right).$$

It can be observed in Equation (15) that *f*_d_r_ depends on the ratio between *L*_2_ and *L*_r_. Nevertheless, if *L*_2_ << *L*_r_ the frequency *f*_d_r_ tends to the resonant frequency *f*_r_ of the electromechanical resonator. A numerical analysis that allows the calculation of the parameters *f*_d_r_ and *τ*_r_ of the complete system, is also reported in [21]. The results can be directly compared with Figure 8, the values of the parameters of the BVD model used in the numerical analysis being the same. Also in that case, good agreement between the values of *f*_d_r_ predicted from Equation (15) and the numerical results have been obtained, with a maximum deviation within 3 ppm for *L*_2_ up to 10 µH.

### 3.4. Effect of Parasitic Capacitance at the Primary Coil on Coil-Coupled Capacitance Sensors

When the proposed techniques are transferred into real electronic circuits, unavoidable nonidealities result in a lumped parasitic capacitance *C*_P_ that appears in parallel to *L*_1_. The parasitic capacitance *C*_P_ is mainly composed of the parasitic capacitance of the inductor *L*_1_, the capacitance of the connections, and the input capacitance of the electronic interface.

The effect of *C*_P_ is now evaluated, firstly, considering the case of the RSU with the capacitance sensor, extending the treatment of Section 3.2.

**Figure 10:**
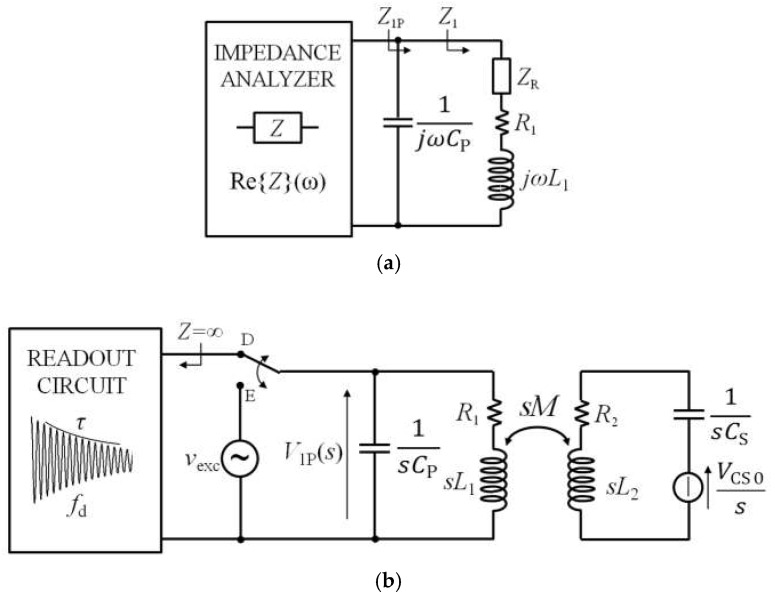
(**a**) Block diagram of the interrogation system with equivalent circuit of the impedance *Z*_1P_ for the technique based on impedance measurements applied to a coil-coupled capacitance sensor; (**b**) block diagram of the interrogation system with equivalent circuit in the Laplace domain to derive *V*_P1_(*s*) during the detection phase of the time-gated technique applied to a coil-coupled capacitance sensor.

With reference to Figure 10a, the real part of the impedance at the primary coil becomes

(16) Re{Z1P}=Re{(R1+jωL1+ω2k2L1L2R2+jωL2+1jωCS)1jωCPR1+jωL1+ω2k2L1L2R2+jωL2+1jωCS+1jωCP}.

As discussed in [23], with *C*_P_ ≠ 0, Equation (16) no longer allows extraction of *f*_S_ and *Q*_S_ independently from the coupling factor *k*, which now is in the expression of *Z*_1P_ and affects Re{*Z*_1P_}, not only as a scaling factor. In particular, it has been shown by a numerical analysis of Equation (16) that Re{*Z*_1P_} has two maxima, corresponding, respectively, to a primary resonance near *f*_S_ and a secondary resonance near $f_{P}=\;1/\;\left(2\pi \sqrt{L_{1}C_{P}}\right)$. Both the frequencies of the maxima and the trend of Re{*Z*_1P_} are influenced by the coupling factor *k* [23].

Considering now the time-gated technique, the voltage *v*_1P_(*t*) at the primary coil in the detection phase can be obtained from the circuit of Figure 10b. Adopting the same approach as for the case of *C*_P_ = 0, it will be assumed that all the reactive elements, except the capacitor *C*_S_, have zero initial conditions at *t* = 0. Consequently, the voltage *V*_1P_(s) can be expressed in the Laplace domain as

(17) $$V_{1P}\left(s\right)=\frac{N\left(s\right)}{D\left(s\right)}=\;k\sqrt{\frac{L_{1}}{L_{2}}}\frac{sV_{\mathrm{CS}0}C_{S}L_{2}}{s^{4}C_{S}C_{P}L_{1}L_{2}\left(1-k^{2}\right)+s^{3}C_{S}C_{P}\left(L_{1}R_{2}+L_{2}R_{1}\right)+s^{2}\left(C_{S}L_{2}+C_{P}L_{1}+C_{S}C_{P}R_{1}R_{2}\right)+s\left(C_{S}R_{2}+C_{P}R_{1}\right)+1},$$

where *V*_CS0_ is the voltage across *C*_S_ at *t* = 0. From Equation (17), it can be seen that *k*, besides acting as a scaling factor, also features in the coefficient of fourth degree in the polynomial *D*(*s*). Consequently, it is expected that the complex frequencies are dependent on *k*. Taking the inverse Laplace transform of Equation (17), it results that the expression of *v*_1P_(*t*) is composed of the sum of two damped sinusoids as

(18) $$v_{1P}\left(t\right)=A_{1}e^{-\frac{t}{\tau_{d1}}}\cos\left(2\pi f_{d1}t-\theta_{1}\right)+A_{2}e^{-\frac{t}{\tau_{d2}}}\cos\left(2\pi f_{d2}t-\theta_{2}\right),$$

where *A*_1_ and *A*_2_ are amplitude coefficients and *θ*_1_ and *θ*_2_ are phase angles that depend on the parameters of the circuit and the initial conditions. The frequencies *f*_d1_ and *f*_d2_ and the decay times, *τ*_d1_ and *τ*_d2_ are obtained by the complex conjugate solutions *p*_1,2_ = 1/*τ*_d1_ ± j2π*f*_d1_ and *p*_3,4_ = 1/*τ*_d2_ ± j2π*f*_d2_ of *D*(*s*) = 0.

From the values of *p*_1,2_ and *p*_3,4_, it can be demonstrated that *f*_d1_ is close to *f*_P_, while *f*_d2_ is close to *f*_S_, but both *f*_d1_ and *f*_d2_ are dependent on *k*. For *R*_2_ sufficiently smaller than *R*_1_, a decay time *τ*_d2_ larger than *τ*_d1_ can be obtained. In this condition, in *v*_1P_(*t*) the damped sinusoid at *f*_d1_ falls off more rapidly than that at *f*_d2_, and it becomes negligible as time elapses. Importantly, since *f*_d2_ depends on *k*, the distance-independent operation of the case *C*_P_ = 0 is now lost.

The dependence of the readout frequency on the coupling factor *k*, introduced by the parasitic capacitance *C*_P_, on both the proposed techniques, is investigated by numerical analysis. For the RSU and CL_1_, the following sample values, which represent real conditions well, have been considered: *L*_2_ = 8 µH, *C*_S_ = 100 pF, *R*_2_ = 3 Ω, *L*_1_ = *L*_2_, and *R*_1_ = 10 Ω. For the impedance technique, the frequency *f*_SP_ has been calculated from the expression of Re{*Z*_1P_}, adopting the definitions in Equation (5). For the time-gated technique, *f*_SP_ has been calculated from *f*_d2_ and *τ*_d2_, derived from the numerical solution of *D*(*s*) = 0, adopting the definitions in Equation (8).

**Figure 11:**
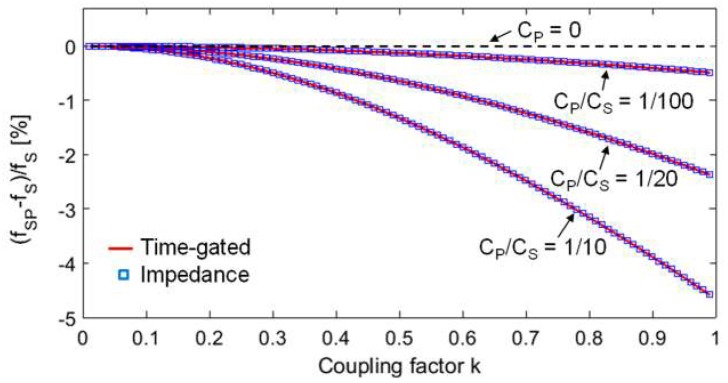
Comparison of the (*f*_SP_ − *f*_S_)/*f*_S_ obtained from the two techniques as a function of *k* for three different values of the ratio *C*_P_*/C*_S_*.* The exact value of *f*_S_ without the parasitic capacitance, i.e., *C*_P_ = 0, is *f*_S_ = 5.626977 MHz.

Figure 11 compares the obtained relative deviation (*f*_SP_ − *f*_S_)/*f*_S_ as a function of the coupling factor *k* for three different values of *C*_P_*/C*_S_. For the considered values of the parameters, *C*_P_ ranges from 1 pF to 10 pF. As it can be observed, (*f*_SP_ − *f*_S_)/*f*_S_ deviates from zero, corresponding to *C*_P_ = 0. The deviation increases for increasing *k* of an amount that augments with *C*_P_*/C*_S_. Noticeably, both the techniques are equally affected by the inaccuracies introduced by *C*_P_, in terms of the dependence of the readout frequency on *k*. These results demonstrate that *C*_P_ prevents accurate distance-independent measurements from being obtained.

### 3.5. Effect of Parasitic Capacitance at the Primary Coil on Coil-Coupled Electromechanical Piezoelectric Resonators

**Figure 12:**
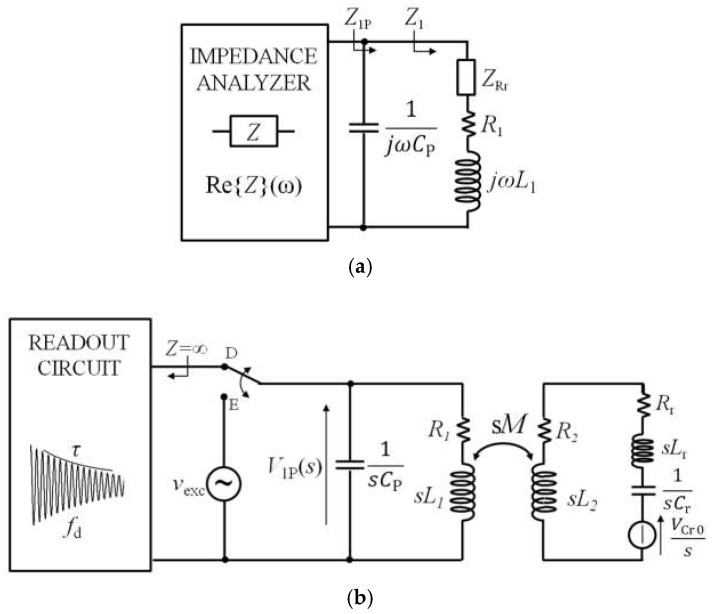
(**a**) Block diagram of the interrogation system with equivalent circuit of the impedance *Z*_1P_ for the technique based on impedance measurements applied to an electromechanical piezoelectric resonator; *Z*_Rr_ represents the reflected impedance of the RSU with electromechanical piezoelectric resonator. (**b**) Block diagram of the interrogation system with equivalent circuit in the Laplace domain to derive *V*_1P_(*s*) during the detection phase of the time-gated technique applied to an electromechanical piezoelectric resonator.

Considering, now, the case with coil-coupled electromechanical piezoelectric resonators, the dependence on *k* due to *C*_P_ can be evaluated by using the same numerical approach as discussed in Section 3.3. The resonant frequency *f*_rP_ can be obtained from numerical analysis of the equivalent circuit in Figure 12a for the frequency-domain technique based on impedance *Z*_1P_, while the equivalent circuit of Figure 12b must be considered for the time-gated technique to determine *V*_1P_(*s*).

In both the equivalent circuits, the impedance of the static capacitance *C*_0_ has been considered high enough to be neglected. For the time-gated technique, *C*_P_ is expected to give rise to an additional damped sinusoid in *v*_1P_(*t*), with a damped frequency related to *C*_P_ resonating with *L*_1_. However, the numerical simulations have demonstrated that this sinusoid fades out more quickly than the damped sinusoid, due to the QCR response.

**Figure 13:**
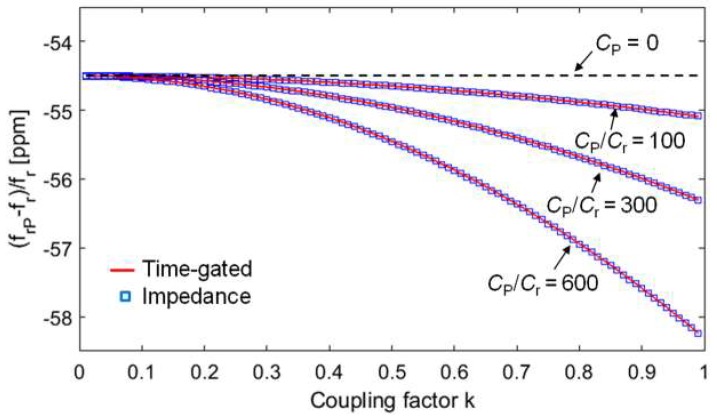
Comparison of the relative deviation (*f*_rP_ − *f*_r_)/*f*_r_ obtained from the time-gated technique and the impedance technique as a function of *k* for three different values of the ratio *C*_P_*/C*_r_.

Considering the same parameter values for the QCR as adopted for the analysis of Figure 8, the obtained relative deviation (*f*_rP_ − *f*_r_)/*f*_r_ as a function of *k* for three different increasing values of the ratio *C*_P_/C_r_, is reported in Figure 13. For the considered values of the parameters, C_P_ ranges from 1.65 pF to 99.2 pF. The baseline, i.e., the dotted curve corresponding to *C*_P_ = 0, is at −54.5 ppm because of *L*_2_, that slightly affects *f*_r2_ and, hence, *f*_rP_, according to Equation (11). As it can be observed, *f*_rP_ has a maximum variation of less than 4 ppm with respect to the baseline. Remarkably, also in this case, the same behaviour with respect to *C*_P_ and *k* is predicted for the two techniques.

The quantitatively negligible dependence of *f*_rP_ on *k* can be ascribed to the fact that the inductive component in the RSU is dominated by *L*_r_. In fact, *L*_r_ is three orders of magnitude larger than *L*_2_, and it is not involved in the coupling between the primary coil and the RSU. This result shows that with coil-coupled electromechanical resonators, such as QCRs, the proposed techniques remain practically independent from the coupling factor *k*, despite a not-negligible *C*_P_.

## 4. Interrogation Techniques and Interface Circuits

### 4.1. Interrogation System Based on the Impedance-Measurement Technique with Parasitic Capacitance Compensation

**Figure 14:**
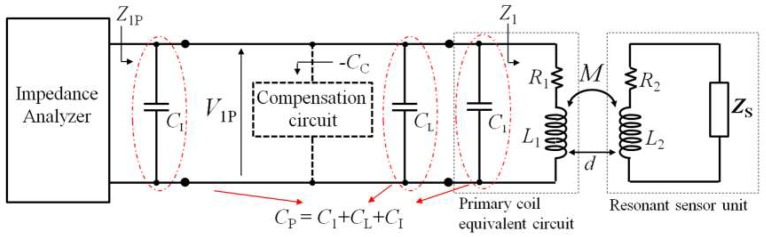
Block diagram of the interrogation system based on impedance measurement technique with parasitic capacitance compensation circuit.

The block diagram of the interrogation system, based on impedance-measurement technique, is reported in Figure 14. The primary coil CL_1_ is connected to the impedance analyzer. The total parasitic capacitance *C*_P_ accounts for the contributions given by the parasitic capacitances of CL_1_, the connections and the equivalent capacitance of the input of the impedance analyzer, represented in Figure 14 with *C*_1_, *C*_L_, and *C*_I_, respectively.

The key idea is that connecting a proper capacitance compensation circuit to the primary coil CL_1_, it is possible to cancel the effects of *C*_P_. The proposed compensation circuit, described in Section 4.3, behaves as an equivalent negative capacitance −*C*_C_. The ideal condition, where *C*_P_ is not present, i.e., *Z*_1P_ = *Z*_1_, can be thus obtained when *C*_C_ = *C*_P_. In the compensated condition, Equation (5) again applies, and *k*-independent measurements of the resonant frequency and quality factor can be obtained by considering the maximum of the real part of the measured impedance.

### 4.2. Interrogation System Based on the Time-gated Technique with Parasitic Capacitance Compensation

**Figure 15:**
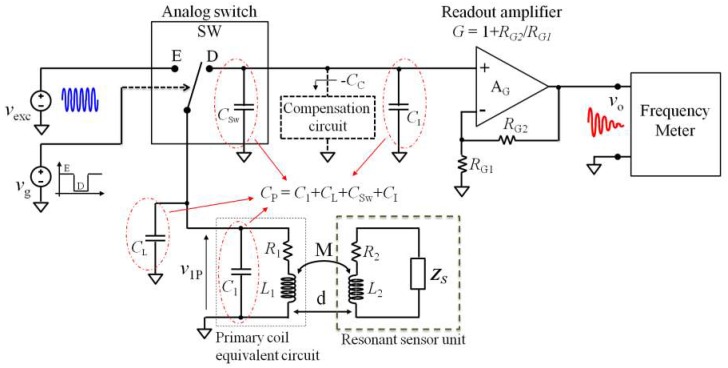
Block diagram of the interrogation system based on of time-gated technique with parasitic capacitance compensation circuit.

The block diagram of the proposed interrogation system based on the time-gated technique is shown in Figure 15. The analog switch SW, controlled by the square-wave gate signal *v*_g_(*t*), alternatively connects the primary coil to the excitation signal *v*_exc_(*t*) and to the high-input impedance readout amplifier A_G_ during the excitation and detection phases, respectively. The noninverting amplifier A_G_, with gain *G*, is based on a high-bandwidth operational amplifier. A frequency meter connected to the output of A_G_ allows measurement of the frequency of the damped sinusoidal signal *v*_O_(*t*).

The total parasitic capacitance *C*_P_ accounts for the contributions of the parasitic capacitances of the primary coil, the connections, the analog switch SW, and the equivalent input capacitance of the amplifier A_G_, represented in Figure 15 with *C*_1_, *C*_L_, *C*_SW_, and *C*_I_, respectively.

Similarly to what was described in Section 4.1, a proper compensation circuit that behaves as an equivalent negative capacitance −*C*_C_ can be introduced to cancel *C*_P_. In the compensated condition, the frequency and decay time of the damped sinusoidal voltage *v*_O_(*t*) return to be unaffected from the coupling factor *k*. In this condition, Equation (8) can be used to extract the resonant frequency and quality factor of the RSU from the measured resonant frequency and decay time of *v*_O_(*t*).

### 4.3. Parasitic Capacitance Compensation Circuit

**Figure 16:**
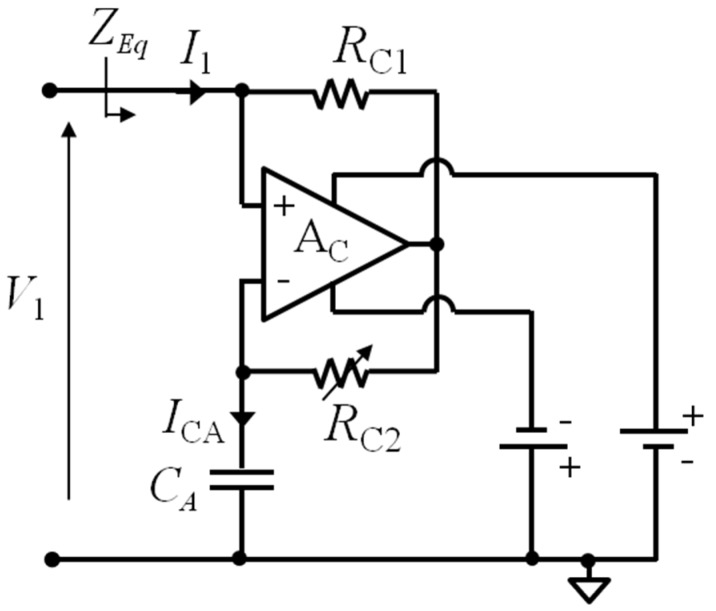
Schematic diagram of the parasitic capacitance compensation circuit.

Figure 16 shows the proposed capacitance compensation circuit. It is based on a high-bandwidth operational amplifier A_C_ operating as a negative impedance converter (NIC) to produce an effective negative capacitance −*C*_C_. The voltage *V*_1_ across CL_1_ is applied across the reference capacitor *C*_A_, thanks to the virtual short circuit at the input of A_C_. The current *I*_CA_ through *C*_A_ is then amplified with gain –*R*_C2_/*R*_C1_, resulting in the current *I*_1_ = −j*ωC*_A_*V*_1_(*R*_C2_/*R*_C1_). The equivalent input impedance *Z_Eq_* = *V*_1_*/I*_1_ is, therefore,

(19) $$Z_{Eq}=\frac{V_{1}}{I_{1}}=\frac{V_{1}}{-\;\frac{j\omega C_{A}V_{1}R_{C2}}{R_{C1}}}=-\frac{R_{C1}}{j\omega C_{A}R_{C2}}=\frac{1}{j\omega \left(-C_{C}\right)}.$$

Then, by taking *C*_A_ and *R*_C1_ as fixed, and making *R*_C2_ variable, the compensation circuit acts as an adjustable negative capacitance, given by

(20) $$-C_{C}=-C_{A}\frac{R_{C2}}{R_{C1}},$$

which can be tuned to compensate and possibly cancel *C*_P_.

## 5. Experimental Results and Discussion

### 5.1. Impedance Measurements with Coil-Coupled Capacitance Sensor and QCR

**Figure 17:**
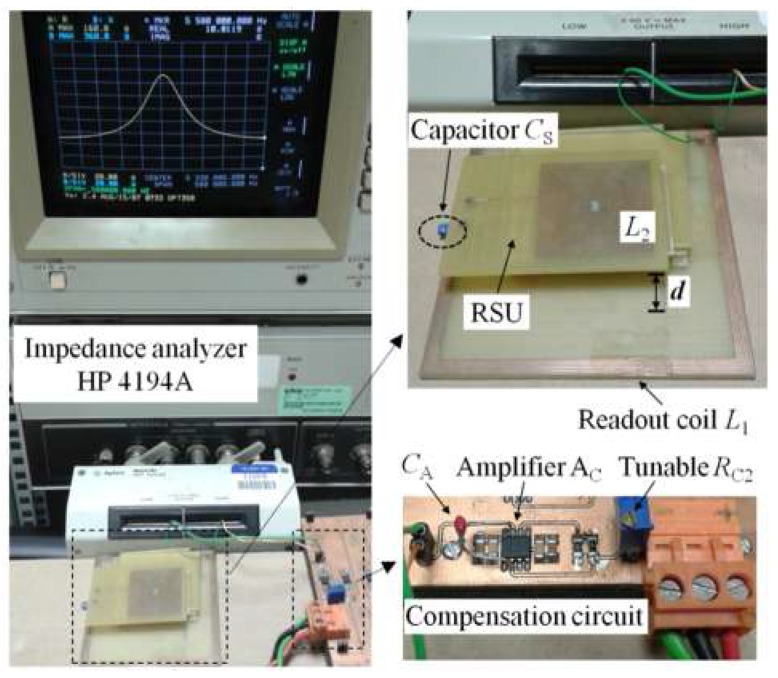
Experimental setup and interrogation system based on impedance-measurement technique with parasitic capacitance compensation.

The experimental setup to test the system, according to the frequency-domain technique based on the block diagram of Figure 14, including the compensation circuit of Figure 16, is shown in Figure 17. The AD8045 (Analog Devices, Norwood, MA, USA) is used for the high-bandwidth operational amplifier A_C_.

For the tests on the capacitance sensor configuration, the RSU is composed of a square planar spiral coil on Printed Circuit Board (PCB) with *L*_2_ = 8.51 µH, *R*_2_ = 3.2 Ω, and a reference capacitor *C*_S_ = 100 pF. According to Equation (1), the resulting resonant frequency and quality factor are *f*_S_ = 5.45 MHz and *Q*_S_ = 91, respectively. A PCB square planar spiral coil has also been used for the primary coil, with *L*_1_ = 8.5 µH and *R*_1_ = 5 Ω. A fixed capacitor *C*_F_ = 22 pF is connected in parallel to the primary coil, in order to set the parasitic capacitance and test the effectiveness of the compensation circuit.

**Figure 18:**
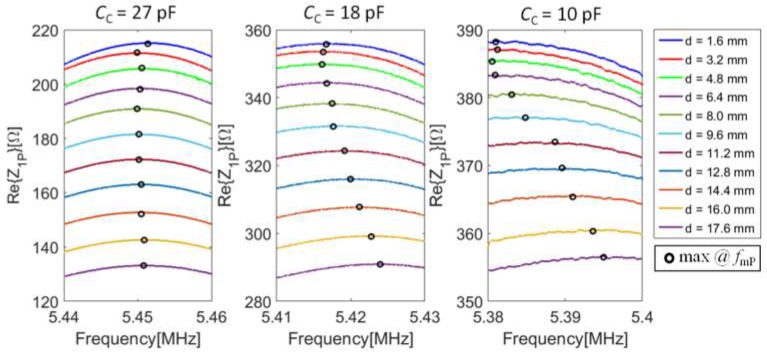
Measured maxima in Re{*Z*_1P_} around *f*_S_ for different values of the compensation *C*_C_, varying the distance *d* between CL_1_ and the RSU. The frequency of the maxima at *f*_mP_ is highlighted with a black circle.

The real part of the impedance *Z*_1P_ versus frequency has been measured at varying interrogation distance *d*, and hence the coupling factor *k*, for different values of the compensation capacitance *C*_C_. The results are shown in Figure 18.

**Figure 19:**
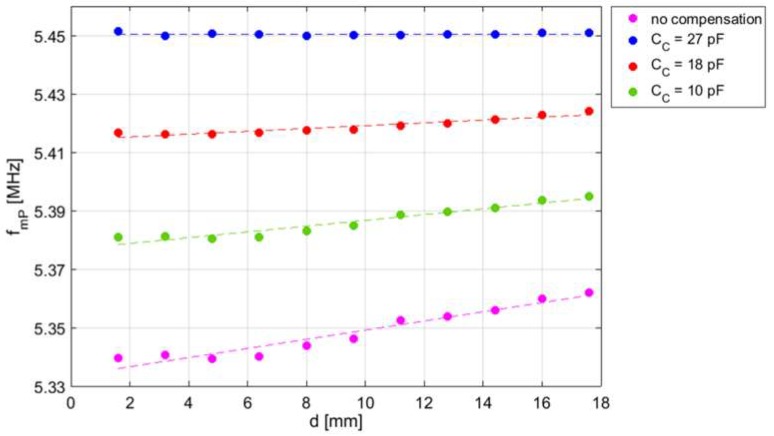
Measured frequency *f*_mP_ as a function of *d* for different values of *C*_C_. The no compensation data are extrapolated from experimental values.

Figure 19 shows the measured frequency *f*_mP_ where the maximum of Re{*Z*_1P_} near *f*_S_ occurs as a function of *d*, for different values of the compensation capacitance *C*_C_. A monotonic decrease of *k* is expected by increasing *d* [29]. It can be observed that by increasing *C*c, the expected undesired effect of the parasitic capacitances described in Section 3.3 decreases. With *C*_C_ = 27 pF, the value of *f*_mP_ becomes independent of *d* over the considered interrogation range of 16 mm, with a residual deviation of *f*_mP_ within 1 kHz, i.e., less than 200 ppm. The obtained value of *C*c = 27 pF, slightly higher than the capacitor *C*_F_ = 22 pF, is ascribed to the presence of an extra capacitance of about 5 pF that concurs to form *C*_P_. The results clearly demonstrate the effectiveness of the compensation technique and circuit.

Under ideal complete compensation condition, the measured *f*_mP_ approaches the unaffected value of *f*_m_, discussed in Section 3.2, over the considered interrogation distance range. Then, for the considered RSU with a *Q*s = 91, a relative deviation |*f*_mP_ – *f*_S_|/*f*_S_ as low as 30 ppm is obtained from Equation (5).

**Figure 20:**
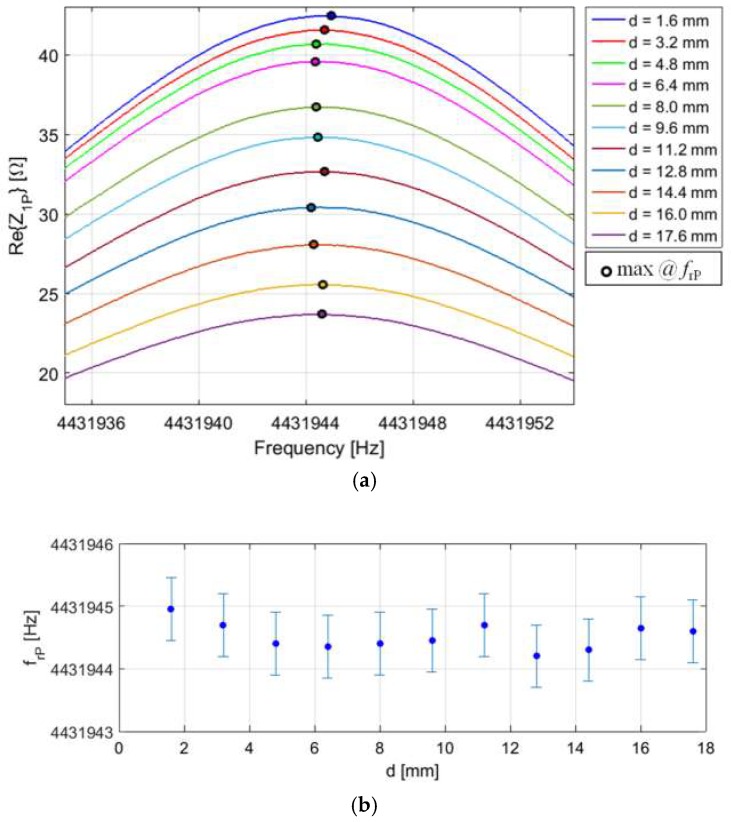
(**a**) Real part of *Z*_1P_ measured around the mechanical resonant frequency *f*_r_ of the quartz crystal resonator (QCR) connected to the primary coil CL_1_ for different distances *d*. The frequency of the maxima at *f*_rP_ is highlighted with a black circle. (**b**) Frequency *f*_rP_ as a function of *d*. The error bars report the standard deviations calculated over 5 repeated measurements.

The same setup has been used for tests on coil-coupled electromechanical piezoelectric resonators. An AT-cut QCR with *f*_r_ = 4.432 MHz has been connected to CL_2_. The parameters of the BVD equivalent circuit around *f*r of the adopted QCR are *C*_0_ = 5.72 pF, *R*_r_ = 10.09 Ω, *L*_r_ = 77.98 mH, and *C*r = 16.54 fF. The numerical analysis, discussed in Section 3.4, proves that parasitic capacitances in the order of tens of picofarads introduce negligible dependence of the measured resonant frequency on *k*. For this reason, the compensation circuit is not connected to the primary coil. Figure 20a shows the real part of the impedance *Z*_1P_, measured in the frequency range around *f*_r_ for different values of the interrogation distance *d*. As it can be observed, while the magnitude of the maximum of Re{*Z*_1P_} decreases by increasing *d*, the frequency *f*_rP_, where the maximum occurs, shows residual variations as low as 1 Hz, i.e., less than 0.3 ppm, in the explored range of *d*, as shown in Figure 20b. This confirms the predicted independence of *f*_rP_ from *d*, and thus from *k*.

### 5.2. Time-Gated Measurements with Coil-Coupled Capacitance Sensor and QCR

**Figure 21:**
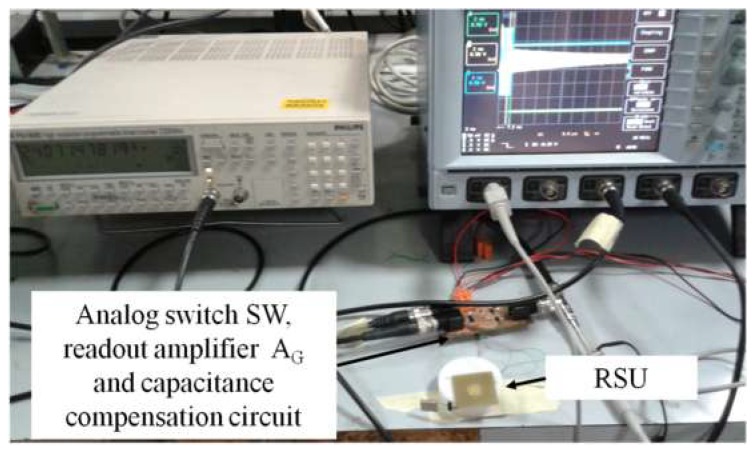
Picture of the experimental setup developed to implement the time-gated technique.

**Figure 22:**
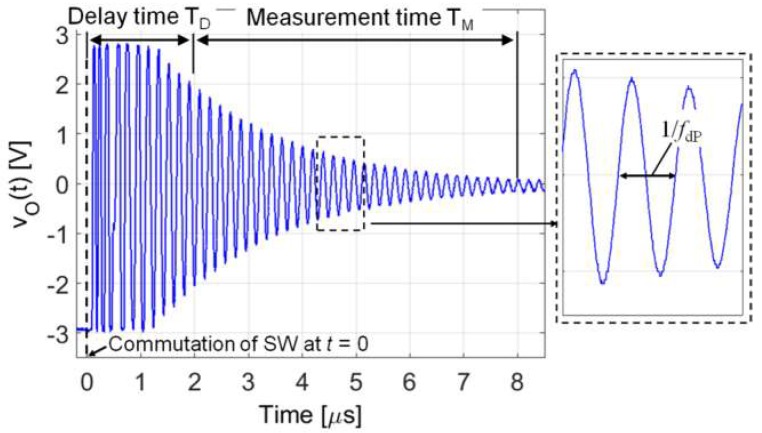
Measured output signal *v*_O_(t) during the detection phase. Indications of the adopted delay time *T*_D_ and measurement time *T*_M_ are reported.

Figure 21 shows the experimental setup used to test the interrogation system based on the time-gated technique shown in Figure 15. The excitation and gate signals *v*_exc_(*t*) and *v*_g_(*t*) are generated by two Agilent 3320A waveform generators (Agilent Technologies, Santa Clara, CA, USA). A tailored circuit comprising the analog switch SW (MAX393, Maxim Integrated, San Jose, CA, USA), the parasitic capacitance compensation circuit, and the readout amplifier A_G_ (OPA656, Texas Instruments, Dallas, TX, USA), has been developed. The readout output signal *v*_O_(*t*) has been connected to a high-resolution frequency meter Philips PM6680 (Philips International, Eindhoven, The Netherlands). The frequency meter is configured to perform measurements in a time window of duration *T*_M_, starting after a delay time *T*_D_ from the beginning of the detection phase. The delay time *T*_D_ is used to skip the initial ringing in *v*_O_(*t*) [18,21]. The voltage *v*_O_(*t*) measured during detection phase, and the times *T*_D_ and *T*_M_, are shown in Figure 22.

Firstly, tests have been done on the RSU with coil-coupled capacitance sensor, described in Section 5.1. The RSU has a PCB spiral coil with *L*_2_ = 8.51 µH, *R*_2_ = 3.2 Ω, and a capacitive sensor with *C*_S_ = 100 pF, resulting in a resonant frequency *f*_S_ = 5.45 MHz. The same PCB spiral coil described in Section 5.1, with *L*_1_ = 8.5 µH and *R*_1_ = 5 Ω, has been used as CL_1_. The frequency of the excitation signal *v*_exc_(*t*) is set close to *f*_S_ to improve the transferred signal level.

**Figure 23:**
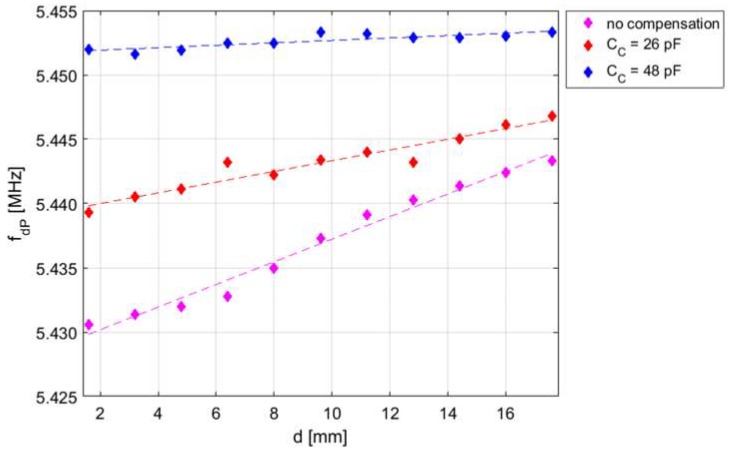
Frequency *f*_dP_ of the damped sinusoid *v*_1P_(*t*) measured as a function of the interrogation distance *d* for different values of the compensation capacitance *C*_C_. A delay time *T*_D_ = 2 µs and a measurement time *T*_M_ = 6 µs have been set in the measurements.

Figure 23 reports the frequency *f*_dP_ of the damped sinusoid *v*_O_(t) during the detection phase, measured at varying *d* for different values of the compensation capacitance *C*_C_. A delay time *T*_D_ = 2 µs and a measurement time *T*_M_ = 6 µs have been chosen for all the measurements. As it can be observed, for the case of compensation of *C*_P_, the dependence of *f*_dP_ on *d* is much reduced with respect to the cases with no or partial compensation. With *C*_C_ ≈ 48 pF, *f*_dP_ has residual variations within 1.5 kHz, i.e., less than 300 ppm, across the explored interrogation range of about 17.6 mm.

Under ideal complete compensation, the measured *f*_dP_ approaches the unaffected value of *f*_d_ discussed in Section 3.2. Then, for the considered RSU with *Q*s = 91, a relative deviation |*f*_dP_ − *f*_S_|/*f*_S_ as low as 15 ppm is obtained from Equation (8).

Then, tests have been run on an RSU made by a coil-coupled 4.432-MHz AT-cut QCR. The capacitance compensation circuit has been kept inactive, due to the predicted independence of *f*_rP_ from *k* for coil-coupled QCR. The frequency *f*_rP_ of the damped sinusoid *v*_O_(*t*) has been measured with varying the interrogation distance *d*.

**Figure 24:**
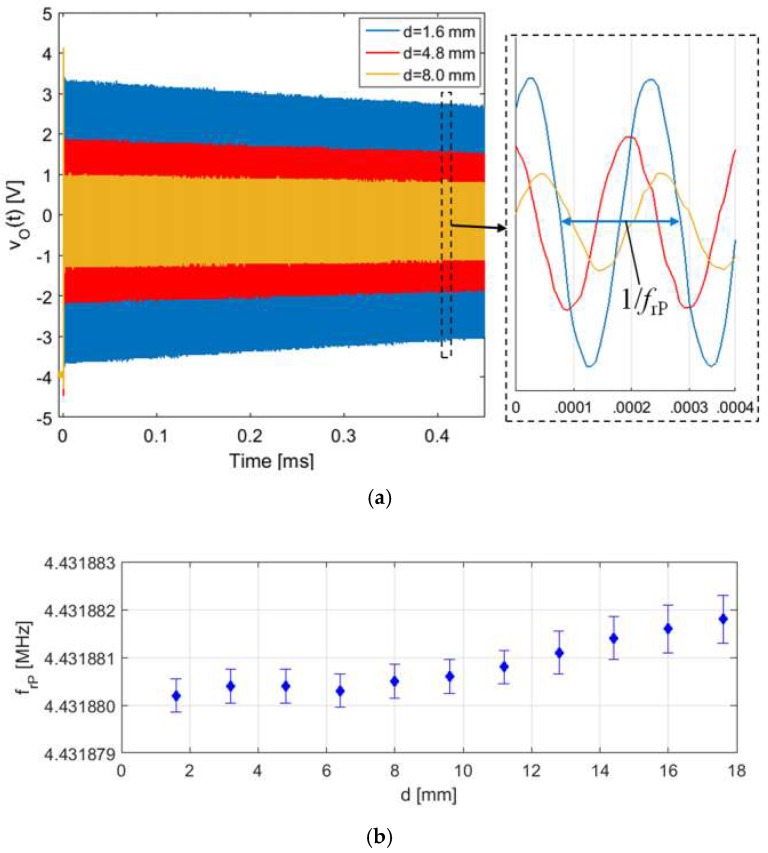
(**a**) Measured output signal *v*_O_(*t*) at the beginning of the detection phase for three different interrogation distances *d*. (**b**) Frequency *f*_rP_ as a function of *d* measured with a delay time *T*_D_ = 5 µs and a measurement time *T*_M_ = 10 ms. The error bars report the standard deviations calculated over 30 repeated measurements.

Figure 24a shows the voltage *v*_O_(*t*) at the beginning of the detection phase for three different interrogation distances *d*. As it can be observed, the magnitude of *v*_O_(*t*) decreases with the increasing *d*, i.e., with decreasing *k*, while, as expected, the frequency *f*_rP_ is unaffected, as shown in Figure 24b. A residual variation of about 1.8 Hz, i.e., less than 0.5 ppm, has been obtained over the explored interrogation distance range of about 17.8 mm. In summary, the experimental results with coil-coupled QCRs show that the total parasitic capacitance *C*_P_ estimated in about 48 pF, causes a negligible variation of the measured frequency *f*_rP_ over the explored interrogation range.

## 6. Conclusions

This work has investigated contactless interrogation techniques and readout circuits for passive sensors, exploiting the electromagnetic coupling between a primary and a secondary coil.

The sensor can be either a capacitive sensor or an electromechanical piezoelectric resonator. With both kinds of sensors, resonance can occur in the secondary circuit that can, therefore, be named resonant sensor unit (RSU). The interrogation of the RSU can be accomplished by techniques operating either in the frequency domain or in the time domain, which are ideally independent of the distance between the primary and secondary coils.

On the other hand, when unavoidable parasitic effects are considered, that combine in a lumped capacitance in parallel to the readout coil, an unwanted dependence of the readout frequency and quality factor on the interrogation distance is introduced, affecting similarly both the frequency- and time-domain techniques. Numerical analysis and experimental tests demonstrate that this dependence is detrimental on the accuracy of the readout frequency of the RSU. The inaccuracies are more relevant for the capacitive sensors, while for electromechanical piezoelectric resonators, the effect is negligible in most cases.

As a solution, an innovative approach has been proposed in which such parasitic capacitance is compensated by a purposely designed electronic circuit that has been prototyped and experimentally verified.

In tests carried out on a capacitive RSU with the proposed compensation circuit applied, a maximum deviation as low as 300 ppm on a resonant frequency of 5.45 MHz has been obtained over an interrogation range of almost 2 cm. This successfully demonstrates the validity of the proposed approach and circuit.

In addition, the experimental results have confirmed that the effect of the input parasitic capacitance is negligible when a coil-coupled piezoelectric quartz crystal resonator is used as the RSU.
